# Supplementary material for: A Critical View of the Application of the APEX Software (Aqueous Photochemistry of Environmentally-Occurring Xenobiotics) to Predict Photoreaction Kinetics in Surface Freshwaters
Source: Molecules. 2019 Dec 18;25(1):9. doi: 10.3390/molecules25010009 (PMC7017383; doi:10.3390/molecules25010009)
Supplement: Supplementary file 1 [file molecules-25-00009-s001.zip › 3.2.4_gcc-4.4.0.zip!/3.2.4_gcc-4.4.0/3.2.4_gcc-4.4.0/doc/octave/html/interpreter/Mathematical-Considerations.html]

Mathematical Considerations - Untitled


Previous: Return Types of Operators and Functions,
Up: Operators and Functions


---

##### 21.1.4.3 Mathematical Considerations

The attempt has been made to make sparse matrices behave in exactly the
same manner as there full counterparts. However, there are certain differences
and especially differences with other products sparse implementations.

Firstly, the "./" and ".^" operators must be used with care. Consider what
the examples

```
       s = speye (4);
       a1 = s .^ 2;
       a2 = s .^ s;
       a3 = s .^ -2;
       a4 = s ./ 2;
       a5 = 2 ./ s;
       a6 = s ./ s;
```

will give. The first example of s raised to the power of 2 causes
no problems. However s raised element-wise to itself involves a
large number of terms `0 .^ 0` which is 1. There s `.^` s is a full matrix.

Likewise s `.^ -2` involves terms like `0 .^ -2` which
is infinity, and so s `.^ -2` is equally a full matrix.

For the "./" operator s `./ 2` has no problems, but
`2 ./` s involves a large number of infinity terms as well
and is equally a full matrix. The case of s `./` s
involves terms like `0 ./ 0` which is a `NaN` and so this
is equally a full matrix with the zero elements of s filled with
`NaN` values.

The above behavior is consistent with full matrices, but is not
consistent with sparse implementations in other products.

A particular problem of sparse matrices comes about due to the fact that
as the zeros are not stored, the sign-bit of these zeros is equally not
stored. In certain cases the sign-bit of zero is important. For example

```
      a = 0 ./ [-1, 1; 1, -1];
      b = 1 ./ a
      => -Inf            Inf
          Inf           -Inf
      c = 1 ./ sparse (a)
      =>  Inf            Inf
          Inf            Inf
```

To correct this behavior would mean that zero elements with a negative
sign-bit would need to be stored in the matrix to ensure that their
sign-bit was respected. This is not done at this time, for reasons of
efficiency, and so the user is warned that calculations where the sign-bit
of zero is important must not be done using sparse matrices.

In general any function or operator used on a sparse matrix will
result in a sparse matrix with the same or a larger number of non-zero
elements than the original matrix. This is particularly true for the
important case of sparse matrix factorizations. The usual way to
address this is to reorder the matrix, such that its factorization is
sparser than the factorization of the original matrix. That is the
factorization of `L * U = P * S * Q` has sparser terms `L`
and `U` than the equivalent factorization `L * U = S`.

Several functions are available to reorder depending on the type of the
matrix to be factorized. If the matrix is symmetric positive-definite,
then symamd or csymamd should be used. Otherwise
amd, colamd or ccolamd should be used. For completeness
the reordering functions colperm and randperm are
also available.

See fig:simplematrix, for an example of the structure of a simple
positive definite matrix.

**Figure 21.3: Structure of simple sparse matrix.**

The standard Cholesky factorization of this matrix can be
obtained by the same command that would be used for a full
matrix. This can be visualized with the command
`r = chol(A); spy(r);`.
See fig:simplechol.
The original matrix had
598
non-zero terms, while this Cholesky factorization has
10200,
with only half of the symmetric matrix being stored. This
is a significant level of fill in, and although not an issue
for such a small test case, can represents a large overhead
in working with other sparse matrices.

The appropriate sparsity preserving permutation of the original
matrix is given by symamd and the factorization using this
reordering can be visualized using the command `q = symamd(A);
r = chol(A(q,q)); spy(r)`. This gives
399
non-zero terms which is a significant improvement.

The Cholesky factorization itself can be used to determine the
appropriate sparsity preserving reordering of the matrix during the
factorization, In that case this might be obtained with three return
arguments as r`[r, p, q] = chol(A); spy(r)`.

**Figure 21.4: Structure of the un-permuted Cholesky factorization of the above matrix.**

**Figure 21.5: Structure of the permuted Cholesky factorization of the above matrix.**

In the case of an asymmetric matrix, the appropriate sparsity
preserving permutation is colamd and the factorization using
this reordering can be visualized using the command `q =
colamd(A); [l, u, p] = lu(A(:,q)); spy(l+u)`.

Finally, Octave implicitly reorders the matrix when using the div (/)
and ldiv (\) operators, and so no the user does not need to explicitly
reorder the matrix to maximize performance.

— Loadable Function: p = **amd** (s)  
— Loadable Function: p = **amd** (s, opts)  
> Returns the approximate minimum degree permutation of a matrix. This
> permutation such that the Cholesky factorization of s `(`p`,` p`)` tends to be sparser than the Cholesky factorization of s
> itself. `amd` is typically faster than `symamd` but serves a
> similar purpose.
>
> The optional parameter opts is a structure that controls the
> behavior of `amd`. The fields of these structure are
>
> opts.dense: Determines what `amd` considers to be a dense row or column of the input matrix. Rows or columns with more than `max(16, (dense * sqrt (`n`)` entries, where n is the order of the matrix s, are ignored by `amd` during the calculation of the permutation The value of dense must be a positive scalar and its default value is 10.0 opts.aggressive: If this value is a non zero scalar, then `amd` performs aggressive absorption. The default is not to perform aggressive absorption.
>
> The author of the code itself is Timothy A. Davis (davis@cise.ufl.edu),
> University of Florida (see http://www.cise.ufl.edu/research/sparse/amd).
>
>
>
> **See also:** symamd, colamd.

— Loadable Function: p = **ccolamd** (s)  
— Loadable Function: p = **ccolamd** (s, knobs)  
— Loadable Function: p = **ccolamd** (s, knobs, cmember)  
— Loadable Function: [p, stats] = **ccolamd** (...)  
> Constrained column approximate minimum degree permutation. p `=
> ccolamd (`s`)` returns the column approximate minimum degree permutation
> vector for the sparse matrix s. For a non-symmetric matrix s,
> s `(:,` p`)` tends to have sparser LU factors than s.
> `chol (`s `(:,` p`)' *` s `(:,` p`))` also tends to be
> sparser than `chol (`s`' *` s`)`. p `= ccolamd
> (`s`, 1)` optimizes the ordering for `lu (`s `(:,` p`))`.
> The ordering is followed by a column elimination tree post-ordering.
>
> knobs is an optional one- to five-element input vector, with a default
> value of `[0 10 10 1 0]` if not present or empty. Entries not present
> are set to their defaults.
>
> knobs`(1)`: if nonzero, the ordering is optimized for `lu (S (:, p))`. It will be a poor ordering for `chol (`s `(:,` p`)' *` s `(:,` p`))`. This is the most important knob for ccolamd. knob`(2)`: if s is m-by-n, rows with more than `max (16,` knobs `(2) * sqrt (n))` entries are ignored. knob`(3)`: columns with more than `max (16,` knobs `(3) * sqrt (min (`m`,` n`)))` entries are ignored and ordered last in the output permutation (subject to the cmember constraints). knob`(4)`: if nonzero, aggressive absorption is performed. knob`(5)`: if nonzero, statistics and knobs are printed.
>
> cmember is an optional vector of length n. It defines the constraints
> on the column ordering. If cmember `(j) =` c, then column
> j is in constraint set c (c must be in the range 1 to
> n). In the output permutation p, all columns in set 1 appear
> first, followed by all columns in set 2, and so on. cmember `=
> ones(1,n)` if not present or empty. `ccolamd (`s`, [], 1 :` n`)` returns `1 :` n
>
> p `= ccolamd (`s`)` is about the same as p `=
> colamd (`s`)`. knobs and its default values differ. `colamd`
> always does aggressive absorption, and it finds an ordering suitable for
> both `lu (`s `(:,` p`))` and `chol (`S `(:,` p`)'
> *` s `(:,` p`))`; it cannot optimize its ordering for
> `lu (`s `(:,` p`))` to the extent that
> `ccolamd (`s`, 1)` can.
>
> stats is an optional 20-element output vector that provides data
> about the ordering and the validity of the input matrix s. Ordering
> statistics are in stats `(1 : 3)`. stats `(1)` and
> stats `(2)` are the number of dense or empty rows and columns
> ignored by CCOLAMD and stats `(3)` is the number of garbage
> collections performed on the internal data structure used by CCOLAMD
> (roughly of size `2.2 * nnz (`s`) + 4 *` m `+ 7 *` n
> integers).
>
> stats `(4 : 7)` provide information if CCOLAMD was able to
> continue. The matrix is OK if stats `(4)` is zero, or 1 if
> invalid. stats `(5)` is the rightmost column index that is
> unsorted or contains duplicate entries, or zero if no such column exists.
> stats `(6)` is the last seen duplicate or out-of-order row
> index in the column index given by stats `(5)`, or zero if no
> such row index exists. stats `(7)` is the number of duplicate
> or out-of-order row indices. stats `(8 : 20)` is always zero in
> the current version of CCOLAMD (reserved for future use).
>
> The authors of the code itself are S. Larimore, T. Davis (Uni of Florida)
> and S. Rajamanickam in collaboration with J. Bilbert and E. Ng. Supported
> by the National Science Foundation (DMS-9504974, DMS-9803599, CCR-0203270),
> and a grant from Sandia National Lab. See
> http://www.cise.ufl.edu/research/sparse for ccolamd, csymamd, amd,
> colamd, symamd, and other related orderings.
>
>
>
> **See also:** colamd, csymamd.

— Loadable Function: p = **colamd** (s)  
— Loadable Function: p = **colamd** (s, knobs)  
— Loadable Function: [p, stats] = **colamd** (s)  
— Loadable Function: [p, stats] = **colamd** (s, knobs)  
> Column approximate minimum degree permutation. p `= colamd
> (`s`)` returns the column approximate minimum degree permutation
> vector for the sparse matrix s. For a non-symmetric matrix s,
> s `(:,`p`)` tends to have sparser LU factors than s.
> The Cholesky factorization of s `(:,`p`)' *` s `(:,`p`)` also tends to be sparser than that of s`' *` s.
>
> knobs is an optional one- to three-element input vector. If s is
> m-by-n, then rows with more than `max(16,`knobs`(1)*sqrt(n))` entries
> are ignored. Columns with more than `max(16,knobs(2)*sqrt(min(m,n)))`
> entries are removed prior to ordering, and ordered last in the output
> permutation p. Only completely dense rows or columns are removed
> if knobs `(1)` and knobs `(2)` are < 0, respectively.
> If knobs `(3)` is nonzero, stats and knobs are
> printed. The default is knobs `= [10 10 0]`. Note that
> knobs differs from earlier versions of colamd
>
> stats is an optional 20-element output vector that provides data
> about the ordering and the validity of the input matrix s. Ordering
> statistics are in stats `(1:3)`. stats `(1)` and
> stats `(2)` are the number of dense or empty rows and columns
> ignored by COLAMD and stats `(3)` is the number of garbage
> collections performed on the internal data structure used by COLAMD
> (roughly of size `2.2 * nnz(`s`) + 4 *` m `+ 7 *` n
> integers).
>
> Octave built-in functions are intended to generate valid sparse matrices,
> with no duplicate entries, with ascending row indices of the nonzeros
> in each column, with a non-negative number of entries in each column (!)
> and so on. If a matrix is invalid, then COLAMD may or may not be able
> to continue. If there are duplicate entries (a row index appears two or
> more times in the same column) or if the row indices in a column are out
> of order, then COLAMD can correct these errors by ignoring the duplicate
> entries and sorting each column of its internal copy of the matrix
> s (the input matrix s is not repaired, however). If a matrix
> is invalid in other ways then COLAMD cannot continue, an error message is
> printed, and no output arguments (p or stats) are returned.
> COLAMD is thus a simple way to check a sparse matrix to see if it's
> valid.
>
> stats `(4:7)` provide information if COLAMD was able to
> continue. The matrix is OK if stats `(4)` is zero, or 1 if
> invalid. stats `(5)` is the rightmost column index that is
> unsorted or contains duplicate entries, or zero if no such column exists.
> stats `(6)` is the last seen duplicate or out-of-order row
> index in the column index given by stats `(5)`, or zero if no
> such row index exists. stats `(7)` is the number of duplicate
> or out-of-order row indices. stats `(8:20)` is always zero in
> the current version of COLAMD (reserved for future use).
>
> The ordering is followed by a column elimination tree post-ordering.
>
> The authors of the code itself are Stefan I. Larimore and Timothy A.
> Davis (davis@cise.ufl.edu), University of Florida. The algorithm was
> developed in collaboration with John Gilbert, Xerox PARC, and Esmond
> Ng, Oak Ridge National Laboratory. (see
> http://www.cise.ufl.edu/research/sparse/colamd)
>
>
>
> **See also:** colperm, symamd.

— Function File: p = **colperm** (s)  
> Returns the column permutations such that the columns of
> s `(:,` p`)` are ordered in terms of increase number
> of non-zero elements. If s is symmetric, then p is chosen
> such that s `(`p`,` p`)` orders the rows and
> columns with increasing number of non zeros elements.

— Loadable Function: p = **csymamd** (s)  
— Loadable Function: p = **csymamd** (s, knobs)  
— Loadable Function: p = **csymamd** (s, knobs, cmember)  
— Loadable Function: [p, stats] = **csymamd** (...)  
> For a symmetric positive definite matrix s, returns the permutation
> vector p such that s`(`p`,`p`)` tends to have a
> sparser Cholesky factor than s. Sometimes `csymamd` works well
> for symmetric indefinite matrices too. The matrix s is assumed to
> be symmetric; only the strictly lower triangular part is referenced.
> s must be square. The ordering is followed by an elimination tree
> post-ordering.
>
> knobs is an optional one- to three-element input vector, with a
> default value of `[10 1 0]` if present or empty. Entries not
> present are set to their defaults.
>
> knobs`(1)`: If s is n-by-n, then rows and columns with more than `max(16,`knobs`(1)*sqrt(n))` entries are ignored, and ordered last in the output permutation (subject to the cmember constraints). knobs`(2)`: If nonzero, aggressive absorption is performed. knobs`(3)`: If nonzero, statistics and knobs are printed.
>
> cmember is an optional vector of length n. It defines the constraints
> on the ordering. If cmember`(j) =` s, then row/column j is
> in constraint set c (c must be in the range 1 to n). In the
> output permutation p, rows/columns in set 1 appear first, followed
> by all rows/columns in set 2, and so on. cmember `= ones(1,n)`
> if not present or empty. `csymamd(`s`,[],1:n)` returns `1:n`.
>
> p `= csymamd(`s`)` is about the same as p `=
> symamd(`s`)`. knobs and its default values differ.
>
> stats `(4:7)` provide information if CCOLAMD was able to
> continue. The matrix is OK if stats `(4)` is zero, or 1 if
> invalid. stats `(5)` is the rightmost column index that is
> unsorted or contains duplicate entries, or zero if no such column exists.
> stats `(6)` is the last seen duplicate or out-of-order row
> index in the column index given by stats `(5)`, or zero if no
> such row index exists. stats `(7)` is the number of duplicate
> or out-of-order row indices. stats `(8:20)` is always zero in
> the current version of CCOLAMD (reserved for future use).
>
> The authors of the code itself are S. Larimore, T. Davis (Uni of Florida)
> and S. Rajamanickam in collaboration with J. Bilbert and E. Ng. Supported
> by the National Science Foundation (DMS-9504974, DMS-9803599, CCR-0203270),
> and a grant from Sandia National Lab. See
> http://www.cise.ufl.edu/research/sparse for ccolamd, csymamd, amd,
> colamd, symamd, and other related orderings.
>
>
>
> **See also:** symamd, ccolamd.

— Loadable Function: p = **dmperm** (s)  
— Loadable Function: [p, q, r, s] = **dmperm** (s)  
> Perform a Dulmage-Mendelsohn permutation on the sparse matrix s.
> With a single output argument dmperm performs the row permutations
> p such that s `(`p`,:)` has no zero elements on the
> diagonal.
>
> Called with two or more output arguments, returns the row and column
> permutations, such that s `(`p`,` q`)` is in block
> triangular form. The values of r and s define the boundaries
> of the blocks. If s is square then r `==` s.
>
> The method used is described in: A. Pothen & C.-J. Fan. Computing the block
> triangular form of a sparse matrix. ACM Trans. Math. Software,
> 16(4):303-324, 1990.
>
>
>
> **See also:** colamd, ccolamd.

— Loadable Function: p = **symamd** (s)  
— Loadable Function: p = **symamd** (s, knobs)  
— Loadable Function: [p, stats] = **symamd** (s)  
— Loadable Function: [p, stats] = **symamd** (s, knobs)  
> For a symmetric positive definite matrix s, returns the permutation
> vector p such that s `(`p`,` p`)` tends to have a
> sparser Cholesky factor than s. Sometimes SYMAMD works well for
> symmetric indefinite matrices too. The matrix s is assumed to be
> symmetric; only the strictly lower triangular part is referenced. s
> must be square.
>
> knobs is an optional one- to two-element input vector. If s is
> n-by-n, then rows and columns with more than
> `max(16,`knobs`(1)*sqrt(n))` entries are removed prior to ordering,
> and ordered last in the output permutation p. No rows/columns are
> removed if knobs`(1) < 0`. If knobs `(2)` is nonzero,
> `stats` and knobs are printed. The default is knobs `= [10 0]`. Note that knobs differs from earlier versions of symamd.
>
> stats is an optional 20-element output vector that provides data
> about the ordering and the validity of the input matrix s. Ordering
> statistics are in stats `(1:3)`. stats `(1) =` stats `(2)` is the number of dense or empty rows and columns
> ignored by SYMAMD and stats `(3)` is the number of garbage
> collections performed on the internal data structure used by SYMAMD
> (roughly of size `8.4 * nnz (tril (`s`, -1)) + 9 *` n
> integers).
>
> Octave built-in functions are intended to generate valid sparse matrices,
> with no duplicate entries, with ascending row indices of the nonzeros
> in each column, with a non-negative number of entries in each column (!)
> and so on. If a matrix is invalid, then SYMAMD may or may not be able
> to continue. If there are duplicate entries (a row index appears two or
> more times in the same column) or if the row indices in a column are out
> of order, then SYMAMD can correct these errors by ignoring the duplicate
> entries and sorting each column of its internal copy of the matrix S (the
> input matrix S is not repaired, however). If a matrix is invalid in
> other ways then SYMAMD cannot continue, an error message is printed, and
> no output arguments (p or stats) are returned. SYMAMD is
> thus a simple way to check a sparse matrix to see if it's valid.
>
> stats `(4:7)` provide information if SYMAMD was able to
> continue. The matrix is OK if stats `(4)` is zero, or 1
> if invalid. stats `(5)` is the rightmost column index that
> is unsorted or contains duplicate entries, or zero if no such column
> exists. stats `(6)` is the last seen duplicate or out-of-order
> row index in the column index given by stats `(5)`, or zero
> if no such row index exists. stats `(7)` is the number of
> duplicate or out-of-order row indices. stats `(8:20)` is
> always zero in the current version of SYMAMD (reserved for future use).
>
> The ordering is followed by a column elimination tree post-ordering.
>
> The authors of the code itself are Stefan I. Larimore and Timothy A.
> Davis (davis@cise.ufl.edu), University of Florida. The algorithm was
> developed in collaboration with John Gilbert, Xerox PARC, and Esmond
> Ng, Oak Ridge National Laboratory. (see
> http://www.cise.ufl.edu/research/sparse/colamd)
>
>
>
> **See also:** colperm, colamd.

— Loadable Function: p = **symrcm** (S)  
> Symmetric reverse Cuthill-McKee permutation of S.
> Return a permutation vector p such that
> S `(`p`,` p`)` tends to have its diagonal elements
> closer to the diagonal than S. This is a good preordering for LU
> or Cholesky factorization of matrices that come from 'long, skinny'
> problems. It works for both symmetric and asymmetric S.
>
> The algorithm represents a heuristic approach to the NP-complete
> bandwidth minimization problem. The implementation is based in the
> descriptions found in
>
> E. Cuthill, J. McKee: Reducing the Bandwidth of Sparse Symmetric
> Matrices. Proceedings of the 24th ACM National Conference, 157–172
> 1969, Brandon Press, New Jersey.
>
> Alan George, Joseph W. H. Liu: Computer Solution of Large Sparse
> Positive Definite Systems, Prentice Hall Series in Computational
> Mathematics, ISBN 0-13-165274-5, 1981.
>
>
>
> **See also:** colperm, colamd, symamd.
